# Inhibition of MicroRNA miR-101-3p on prostate cancer progression by regulating Cullin 4B (CUL4B) and PI3K/AKT/mTOR signaling pathways

**DOI:** 10.1080/21655979.2021.1949513

**Published:** 2021-08-01

**Authors:** Zhenhua Gu, Zhixin You, Yucheng Yang, Rui Ding, Meili Wang, Jianming Pu, Jian Chen

**Affiliations:** a Department of Urology, Wuxi Traditional Chinese Medicine Hospital, Wuxi, China; b Department of Urology, Kunshan Second People's Hospital, Kunshan City, China; c Department of Urology, Changshu Hospital Affiliated to Nanjing University of Chinese Medicine, Changshu, China

**Keywords:** miR-101-3p, CUL4B, PI3K/AKT/mTOR, prostate cancer

## Abstract

To probe into the efffects of miR-101-3p via regulating CUL4B within PI3K/AKT/mTOR signaling pathway on progression of prostate cancer (PCA). Western blot and qRT-PCR were adopted to detect CUL4B and miR-101-3p expressions in 75 cases with PCA . The cellular strains of PCA (LNCaP and PC3) were chose as the objects to check the targeting correlation between CUL4B and miR-101-3p through dual-luciferase reporter experiments. LNCaP cells and PC3 cells were randomly divided into the blank group, miR-101-3p mimic group, siRNA negative control (NC) group, CUL4B siRNA group and CUL4B siRNA plus the miR-101-3p inhibitor group. Cellular bioactivity measurement was done via Cell-Light EDU, MTT, Annexin-V-FITC/PI, scratch-heal experiments and invasion tests of Transwell. MiR-101-3p expression was decreased more signally in tumor tissues than in normal tissues adjacent to the cancer. MiR-101-3p inhibited cellular proliferating, migrating and invasion. Nevertheless, it promoted cellular apoptosis, up-regulated apoptotic proteins as well as down-regulated anti-apoptotic proteins. CUL4B siRNA and miR-101-3p simulation were similar in terms of their outcomes. Nonetheless, these results could be reversed through the miR-101-3p inhibitor. Besides, CUL4B siRNA and the simulation halted a serious of PI3K signal in PCA cells. MiR-101-3p expression was down-regulated in PCA patients. CUL4B was upregulated in PCA patients. Moreover, miR-101-3p suppressed cellular invasion, migration, proliferation and led to cellular apoptosis, which might be related to the PI3K/AKT/mTOR signaling pathway suppression. Finally, we found, MiR-101-3P suppressed PCA progression via aiming for CUL4B, which may offer the new molecular target for PCA clinical treatment.

## Graphical abstract

**Figure:**
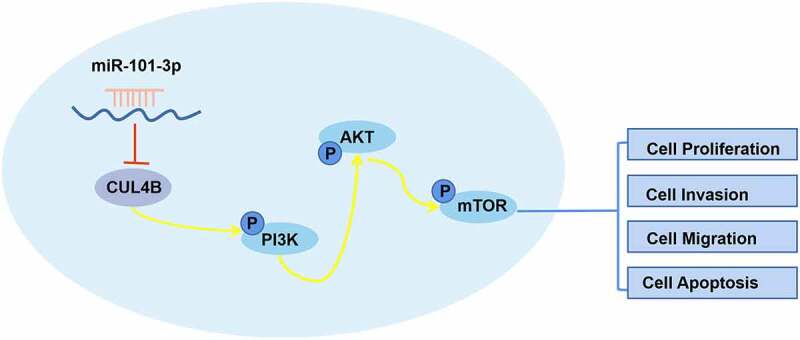

## 1. Introduction

It was estimated that there were more than 1.2 million cases of prostate cancer (PCA) and 359,000 deaths in 2018, which lead to PCA as the second most common tumors among males [1]. Although surgerieswith chemotherapy achieve huge progress, the PCA incidence all over the world rises steadily. Other than that, relapse rate or metastatic rate are relatively high, which leads to a decrease in treatment results and survival rate [2].

The prostate-specific blood test has been used in various stages of prostate cancer management, including screening and the assessment of future risk of prostate cancer development, detection of recurrent disease after local therapy and in the management of advanced disease. However, there were some arguments in terms of the PSA test nowadays. It has been suggested that PSA detection decreases PCA mortality while the test possesses low specificity, causing unnecessary prostate biopsy and over-diagnosis. These outcomes bring about more severe side effects in males [3–6]. Thus, it is pivotal for developing specific biomarker and therapy to explore PCA mechanism.

It is reported that miRNAs, one group of non-coding small and conservative RNAs, can combine with 3ʹUTR in the arm of mRNA to modulate genetic expression [7]. Many miRNAs played a vital role of anti-cancer genes or pro-cancer genes effects in different types of tumor cells, including the cells of PCA. These miRNAs also take part in lots of biological processes such as apoptosis, proliferation as well as metastasis [8]. MiR-101-3p has been reported to function as a tumor suppressor in various types of cancer [9]. One study indicated that over-expressing miR-101-3p inhibited the progress in bladder urothelial [10]. And the glioblastoma metastasis as well as proliferation can be suppressed by miR-101-3p [11]. However, the biological function and underlying mechanisms of miR-101-3p in PCA are largely unknown.

Cullin4B(CUL4B), the compound construction in terms of the E3 ligase in Cullin 4B circle, exerts significant role in protein hydrolysis [12]. It has been reported that the mutation in CUL4B is related to brain malformation [13]. Other studies also found the correlation between CUL4B and the mechanism in AIA [14]. Recently, researchers found that CUL4B has a certain relationship with cancer development and progression including PCA [15–18]. In addition, nowadays much attention has focused on developing targeted therapies that inhibit the PI3K/AKT/mTOR signaling network which is dysregulated in many cancer types. The PI3K/AKT/mTOR pathway regulates cell proliferation, growth, cell size, metabolism, and motility. Component genes of this pathway have been extensively studied and found to be commonly activated in human cancer. Inhibition of this pathway has been shown to lead to regression of human tumors and has been studied in preclinical setup and evaluated in many clinical trials at various levels [19].

In this study, we aim to explore the role of CUL4B and miR-101-3p in PCA cells, as well as the mechanism of miR-101-3p/CUL4B/axis regulating PCA cells, and propose a hypothesis that miR-101-3p targets CUL4B expression can inhibit PCA progression through PI3K/AKT/mTOR signaling pathway.

## 2. Approaches and materials

### 2.1. Ethics statement

This study was approved by the ethics committee in Wuxi Traditional Chinese Medicine Hospital while all patients were informed and then signed the informed papers.

### 2.2. Research subjects

Seventy-five cases of patients with PCA confirmed in Wuxi Traditional Chinese Medicine Hospital from June 2015 to June 2017. Surgically excised tissues were acquired from 75 patients, all the experimental specimens were quickly snap-frozen and kept −150°C for future use. The average age of them were (50.4 ± 6.3) years. The inclusion standard was as followed: (A) over 18 years old, (B) diagnosed as PCA via imaging and pathology tests and (C) without receiving chemotherapy, surgery, radiotherapy before their admitting to the hospital. The exclusion standard was as followed: (A) prostate tumor with a good prognosis, (B) hemopathy, (C) coagulation dysfunction or (D) liver, kidney and heart dysfunction. miR-101-3p and clinicopathological characteristics of prostate cancer shown in Table 1.

**Table 1.** Correlation between miR-101-3p or CUL4B and clinicopathological characteristics of prostate cancer

Characteristics	Group	miR-101-3p expression level			*P*	CUL4B expression level			*P*
		Cases	High (n = 37)	Low (n = 38)		Cases	High (n = 37)	Low (n = 38)	
Age	< 60	27	12	15	0.5253	45	20	25	0.3506
	≥60	48	25	23		30	17	13	
Tumor size (cm)	<5 cm	36	25	11	0.0008	38	13	25	0.0111
	≥5 cm	39	12	27		37	24	13	
Histological grade	Moderate	31	17	14	0.8005	43	23	20	0.4861
	Low	44	20	24		32	14	18	
TNM staging	I+ II	48	19	29	0.0243	50	30	18	0.0036
	III+IV	29	18	9		27	7	20	
Gleason score	<7	29	19	10	0.0339	44	17	27	0.0358
	≥7	46	18	28		31	20	11	
Preoperative PSA (ng/mL)	≥10	49	29	20	0.0286	42	14	28	0.0025
	<10	26	8	18		33	23	10	

### 2.3. Cellular culture

Prostatic epithelial cells (RWPE-1) and PCA cellular system (LNCaP, DU145 and PC3) were taken from USA tissue culture bank (MANASSAS, VA). Culture medium 1640 with 10% fetal bovine serum (Atlanta Biologicals, Atlanta, GA), 1% penicillin (Baomanbio) as well as 1% streptomycin (Baomanbio, Shanghai, Chinese Mainland) from Roswell Park Memorial Institute (RPMI) was adopted to cultivate all cellular systems mentioned above. All the cells grew under 37°C in incubator with 5% CO_2_.

### 2.4. Cellular transfection

LNCaP cells and PC3cells were cultivated in 6-well plates. Then, the small interfering RNA targeting CUL4B, the overexpressed plasmid, miR-101-3p mimic/inhibitor and the corresponding NC were transfected into the cells by liposome. The plasmids mentioned above were styled and projected by Gene Pharma from Shanghai in China. Blank plasmids were used as the control. After mixed the Opti-MEM and plasmids, 2000 liposome reagent (Thermo Fisher Science, Waltham, MA, USA) was adopted for cellular transfection under instructions from the manufacturer. After standing the mixture for 20–30 minutes, cells that were transfected steadily were picked up via Geneticin (Sigma-Aldrich, StLouis, MO, USA).

### 2.5. Dual-luciferase reporter test

The experiment work was grouped into miR-NC plus CUL4B-WT, miR-101-3p MIMICS plus CUL4B-WT, miR-101-3p inhibitor plus CUL4B-WT, miR-NC plus CUL4B-MUT, miR-101-3p MIMICS plus CUL4B-MUT and miR-101-3p inhibitor plus CUL4B-MUT. According to the directions of these manufacturers, our team used 2000 liposome transfection reagent (Thermo Fisher Science) to transfect PC3 cells and LNCaP cells on the exponential phase (1 × 10^6^/ml). After 2 days, dual-luciferase reporter gene detected the luciferase activity from Photinus Pyralis. The luciferase activity from Photinus Pyralis was utilized to represent relative luciferase activity. We repeated these experiments three times.

### 2.6. Real-time reverse transcription PCR (qRT-PCR)

Total RNA from cell and human tissue samples of 76 cases was extracted by the Trizol approach (No. 16,096,020, Thermo Fisher Scientific, Waltham, MA, USA). The specific process is as follows: 1) First, we quickly ground the tissue in liquid nitrogen, transferred it to a 1.5 mL centrifuge tube, added 1 mL of Ltrisol per 100 mg of tissue, and blew evenly with a pipette to fully lyse the tissue; 2) Added 300uL of chloroform to the tube, and shook it upside down vigorously for 30 s. After left it at room temperature for 5 minutes, centrifuged it at 12000 rpm/min, 4°C for 10 min. It can be seen that there were three different layers in the centrifuge tube, and slowly placed it on ice; 3) Carefully sucked colorless aqueous phase on the upper layer to transfer to the Rnase-free 1.5 mL EP tube, added an equal volume of isopropanol, and placed it at room temperature for 5 min, 12000 rpm/min to centrifuge at 4°C for 10 min. A small amount of precipitation can be seen at the bottom; 4) Carefully discarded the supernatant and used 75% to wash 2 times with alcohol, 1 mL each time, gently blew up the precipitation with a pipette to centrifuged at 12000 rpm/min at 4°C for 5 min; 5) Let the ultra-clean workbench 1 to dry it for 5–10 min, added an appropriate amount of DEPC water to dissolve, and stored at −20°C or −80°C for later use. 5cDNAg was taken to be reversely transcribed to cDNA under the guide of qRT-PCR kits from ABI Company in NY state. The inter reference of relative miR-101-3p expression and relative CUL4B expression was U6 and β-Actin, respectively. Takara bio-tech limited company designed and synthesized U6, miR-101-3p, β-Actin and CUL4B primers(Dalian, Liaoning, China) (Table 1). The 2-ΔΔCt approach was adopted to calculate fold changes. The formula was as follows: ΔΔCT = ΔCt (the experiment ones)-ΔCt (the control ones), ΔCt = CtmiRNA-CtU6. CT was amplification numbers till when the intensity of fluorescence RT reached the setting doorsill. At that time the logarithmic amplification appeared. Primer sequences for RT-qPCR (Table 2).

**Table 2.** Primer sequences for RT-qPCR

Gene	Primer sequences
miR-101-3p	Forward:5ʹ-ACGGGCGAGCTACAGTACTGTG-3ʹ,
	Reverse: 5ʹ-CCAGTGCAGGGTCCGAGGTA-3ʹ;
CUL4B	Forward:5ʹ-GGGAAAGGAATGGTGAA-3ʹ
	Reverse:5ʹ-TGCATAGAGCCGGTTAG-3ʹ
U6	Forward:5ʹ-CGCTTCGGCAGCACATATAC-3ʹ,
	Reverse: 5ʹ-TTCACGAATTTGCGTGTCAT-3ʹ.
ʹ-Actin	Forward:5ʹ-ATAGCACAGCCTGGATAGCAACGTAC-3ʹ,
	Reverse:5ʹ-CACCTTCTACAATGAGCTGCGTGTG-3ʹ;

### 2.7. Western blotting

Cells from PCA were collected two days after transfection. Total protein was collected after 10-minute dissociation. After quantifying through BCA, the total protein was set in lanes with 20 µg each. Total proteins were deviated under SDS-PAGE using 100 V. Afterward, those stuff were transferred to cellulose nitrate membranes for 120 minutes under 30 mA condition. Later, the membrane was incubated with the primary antibodies: Bcl2(1:1000, ab59348), CUL4B (1:1000, ab76470), AKT(1:500, ab8805), Bax(1:1000, ab32503), PI3K(1:1000, ab151549), p-mTOR(1:2000, ab137133), mTOR(1:2000, ab2732), p-AKT(1:1000, ab38449), p-PI3K(1:1000, ab182651), Caspase-3(1:1000, ab2303). Our crew bought all the monoclonal antibodies from the Abcam company (Cambridge, UK). Then, the membrane and anti-rabbit antibodies from the goats in terms of IgGL&H were incubated for two hours under the room temperature (horseradish peroxidase-conjugated, ab205718, 1:10,000). We developed fluorescence detection kits for ECL. The Image J software was employed to set the gray scale of aim protein band as well as the band of β-Actin used as the relative level in terms of proteins.

### 2.8. Cellular proliferation measurement

Those cells on the exponential phase and their suspension were placed in environment of 37°C and 5% CO_2_. Then, 10 μL blue thiazole bromide was added for 12, 24,36 and 48 hours. 100 μL DMSO was added into supernatant after centrifugation. The absorbance was estimated under 450 nm and our crew repeated these steps three times. Besides, we also used EDU cellular proliferation detection kits to verify these data. One day after transfection, EDU (50μ/L) was adopted for incubation within 2 hours. Apollo dye marked EDU positive cells (red, 653 nm). DAPI was employed to mark cell nuclei (blue color, 350 nm). The number of EDU positive cells and the total cells were calculated using image pro plus software. Proliferation rate was calculated by number of EDU positive cells dividing total cell number.

### 2.9. Cellular migration tests

Cells were inoculated in the 6-well plate. The P10 pipette tip was used for the artificial wound after cellular adherence(0 h). After one day, we took the photo of cells under an inverted microscope (100×). The software ImagePro Plus 6.2 was used to calculate the percentage number of healing within the scratch area in 24 hours compared with 0 hour. The experiments were repeated three times.

### 2.10. Transwell invasion tests

We added Matt Riegel gel(100 μL) into serum-free culture medium prepared with ice. The whole system was added Transwell tiny chamber under 37°C, conserving for 30 minutes. 100 μL cellular suspension and 200 μL serum-free culture fluid were added into Transwell upper chamber. Transwell lower chamber and complete culture medium with 500 μL 0.05% fetal bovine serum were incubated together for two days with 5% CO_2_. The upper chamber was fixed for half an hour via 4% paraformaldehyde and was dyed for 1 minute via crystal violet. Ethanol was adopted to dehydrate this system by gradient. The xylene was used to clear the samples. Polyester films were cut from the upper room base. Our team placed neutral resin seals on the PPT. We counted six fields randomly. The average field was chosen under high magnification (×400). We repeated these experiments three times.

### 2.11. Annexin-V-FITC/PI dye

Cells were digested by 0.25% trypsin. Cell collection was done after centrifugation and remove the supernatant. PBS liquid was employed to clean cells three times followed by centrifugation. With the help of Annexin series of cellular apoptosis estimation kits (K201-100, Biovision), the right-lower quadrant was the site for early apoptotic cells. The right-upper quadrant was the place for late apoptotic cells. The left-upper quadrant was the location for dead apoptotic cells. Apoptotic rate (%) was equal to the sum of early apoptosis percentage and late apoptosis percentage.

### 2.12. Statistical analysis

All the analyses were performed by version 21.0 SPSS (Inc, Chicago, Illinois). The measurement data were expressed in the form of mean ± standard deviation (X± SD) and were analyzed through t-test between different groups. We utilized one-way ANOVA to understand differences among groups. Turkish HSD tests were used to test results between different groups. *P value < 0.05, **P value < 0.01 and ***P value < 0.001 were regarded statistically significant.

## 3. Results

Here, we aim to explore the role of CUL4B and miR-101-3p in PCA cells, and the regulatory mechanism of miR-101-3p/CUL4B/axis in PCA cells. We conducted a series of in vitro experiments and found that miR-101-3p can inhibit PCA progression through PI3K/AKT/mTOR signaling pathway by targeting and regulating CUL4B expression. Therefore, our data is the first to study the function and mechanism of CUL4B and miR-101-3p in PCA, providing new insights into PCA pathogenesis.

### 3.1. The expression of CUL4B and miR-101-3p of PCA patients

**Figure 1.**
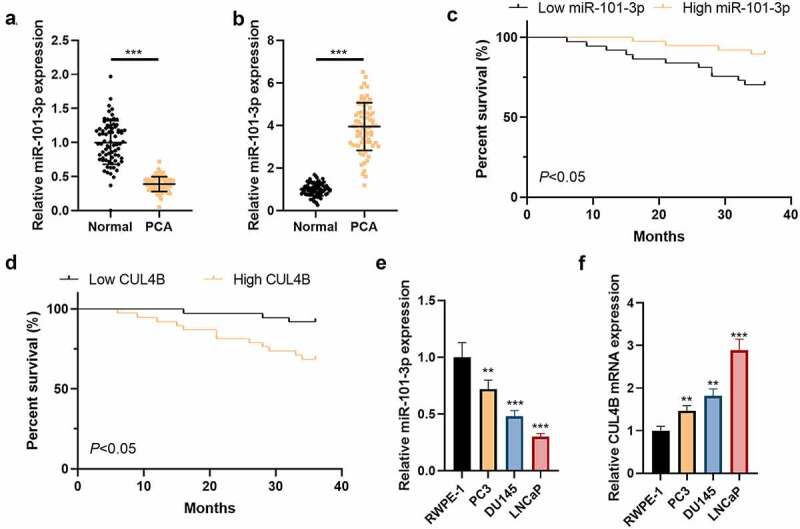
CUL4B as well as miR-101-3p expression among Pca tissues plus cells (a) MiR-101-3p expression among Pca tissues was analyzed through qRT-PCR. (b) The miR-101-3p expression from normal prostatic epithelial cells was measured through qRT-PCR. (c) Kaplan–Meier was used to analyze the relationship between miR-101-3p expression and the survival and prognosis of esophageal cancer patients. (d) Kaplan–Meier was used to analyze the relationship between CUL4B expression and the survival and prognosis of esophageal cancer patients. (e) miRNA expression of CUL4B from Pca tissues was measured through qRT-PCR. (f) mRNA expression of CUL4B in normal prostatic epithelial cells and Pca cells were tested through qRT-PCR. **P < 0.01, ***P < 0.001

We detected CUL4B as well as miR-101-3p expression in PCA patients through qRT-PCR. The results showed that less miR-101-3p expressed in PCA tissues as well as PCA cells compared with those from tissues adjacent to tumors as well as prostatic epithelial cells (*P* < 0.01 or *P* < 0.001, Figure 1a and b). In addition, we tested the correlation between miR-101-3p and CUL4B and clinicopathological characteristics of PCA. As shown in Table 2, miR-101-3p and CUL4B expressions are highly correlated with PCA TNM staging, Gleason scores of tumor size, and preoperative PSA; in addition, based on the median value of the relative miR-101-3p and CUL4B expression levels, PCA patients were divided into two groups: low expression group and high expression group. Kaplan–Meier was employed to analyze the effects of miR-101-3p and CUL4B expression levels on the survival and prognosis of PCA patients. The results showed: patients with low miR-101-3p expression had worse prognosis than that of patients with high, but the prognosis of patients with high CUL4B expression was worse than that of patients with low (P < 0.01, Figure 1c and d). The outcomes showed that more CUL4B conveyed in PCA tissues as well as PCA cells compared with those from tissues adjacent to tumors as well as prostatic epithelial cells (*P* < 0.01 or *P* < 0.001, Figure 1e and f).

### 3.2. MiR-101-3p targeted CUL4B

**Figure 2.**
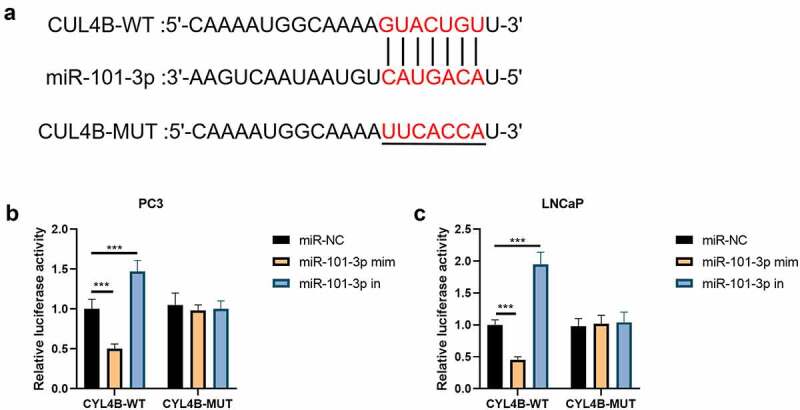
miR-101-3p has combination correlation with CUL4B. (a) We found combination sites between CUL4B and miR-101-3p. (b–c) Dual-luciferase reporter experiments showed that CUL4B from LNCaP cells and PC-3 cells was aimed by miR-101-3p. ***P < 0.001

In Figure 2a, we found that the combination site of miR-101-3p was located in CUL4B-3ʹUTR through website Targetscan (http://www.targetscan.org/vert_72/). Then, the luciferase reporting test implied that there were no significant differences between miR-NC groups, miR-101-3p simulation groups and miR-101-3p inhibitor groups in terms of CUL4B-mutation groups. The simulation suppressed corresponding luciferase action in LNCaP cells and PC3 cells while its inhibitors helped increase the relative luciferase action among LNCaP as well as PC3 cells in terms of CUL4B-WT groups (*P* < 0.001, Figure 2b–c)

### 3.3. MiR −101-3p had an influence in CUL4B expression in human PCA cells

**Figure 3.**
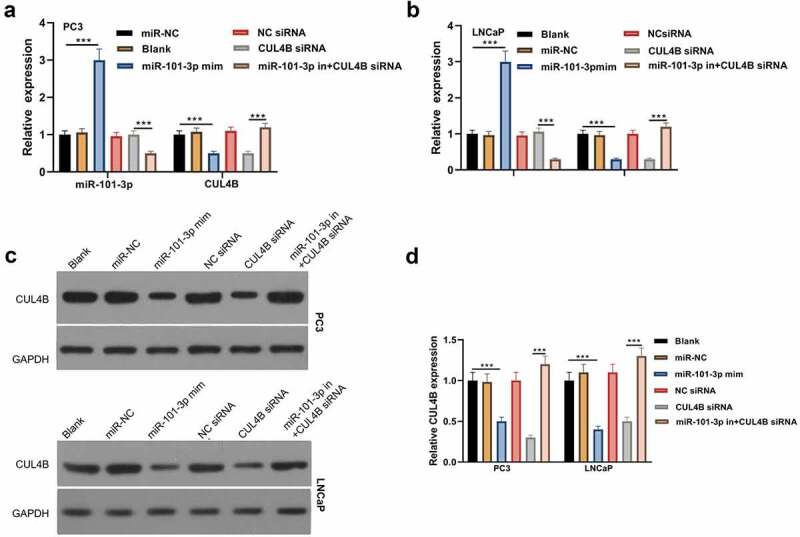
miR-101-3p effects on CUL4B in human Pca cells (a–b) qRT-PCR was adopted to detect CUL4B and miR-101-3p expression among LNCaP as well as PC3 cells. (c–d) Protein CUL4B that expressed in LNCaP as well as PC3 cells was estimated through WB. ***P < 0.001

From Figure 3a and b, we found that miR101-3p level was increased after miR101-3p mimic treatment and CUL4B level was decreased after CUL4B siRNA treatment both in PC3 and LNCaP cells (*P* < 0.001). Although CUL4B siRNA did not influences the level of miR101-3p, miR101-3p inhibitor decreased the miR101-3p expression significantly (*P* < 0.001). Other than that, miR101-3p mimic decreased the level of CUL4B obviously (*P* < 0.001). And compared with the CUL4B siRNA group, miR101-3p inhibitor dramatically increased the level of CUL4B (*P* < 0.001). We were able to find that the change of CLUB protein expression was consistent with the CLUB mRNA from Figure 3c and d.

### 3.4. MiR-101-3p inhibited PCA cellular proliferation through targeting CUL4B

**Figure 4.**
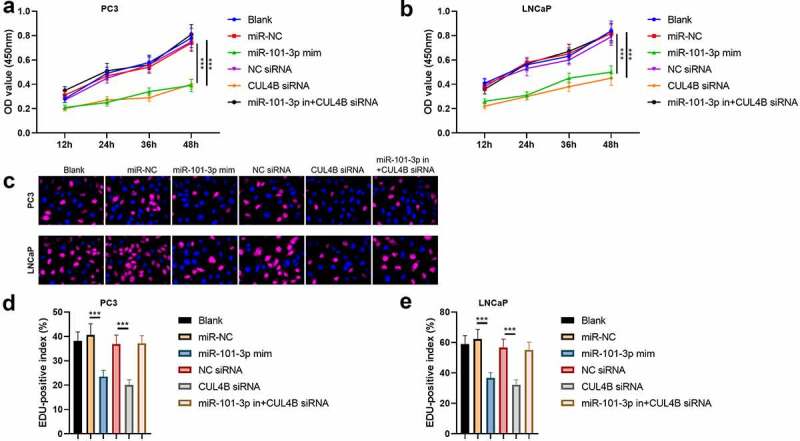
Pca cellular proliferation was suppressed through miR-101-3P aiming for CUL4B. (a–b) MTT approaches were used to detect LNCaP and PC3 cellular proliferation from different groups. C The inverted fluorescence microscope observed growth situation of LNCaP and PC3 cells. Cellular nuclei were dyed through DAPI staining (blue). Red dye meant that cells were proliferating. (d–e) PC3 cells were compared with LNCaP cells in terms of EDU positive index. The positive number in terms of EDU was shown in the form of the percentage number, which was a positive number/total cellular number in details. ***P < 0.001

According to the MTT assay, we found that OD value in miR-101-3p mimic group and CUL4B siRNA group were significantly lower than other groups both in PC3 and LNCaP cells (*P* < 0.001), which means that miR-101-3p and CUL4B siRNA inhibited the cellular proliferation (Figure 4a and b). On the other hand, miR-101-3p mimic decreased positive number in terms of EDU. And miR-101-3p inhibitor reversed EDU positive index repression caused by CUL4B siRNA both in PC3 and LNCaP cells (*P* < 0.001, Figure 4c–e).

### 3.5. MiR-101-3p induced PCA cellular apoptosis via targeting CUL4B

**Figure 5.**
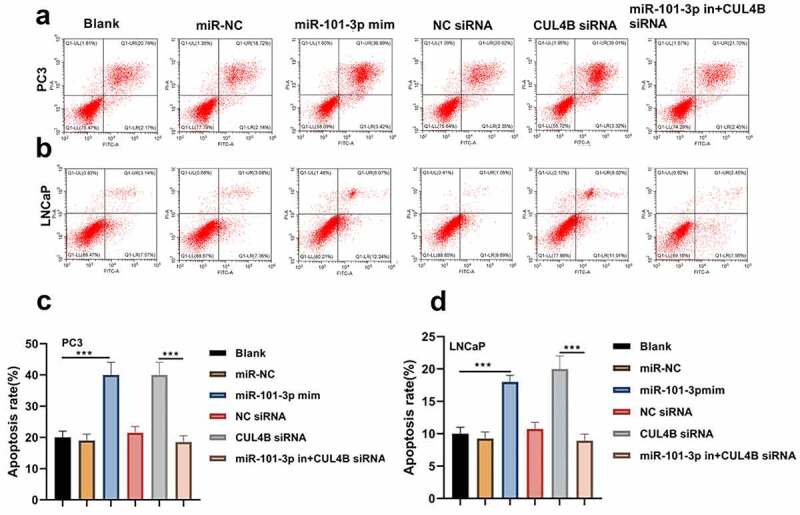
Cellular apoptosis situation in LNCaP and PC3 cells (a-d) We employed Annexin V-FITC/PI dye to display cellular apoptosis situation in LNCaP and PC3 cells. ***P < 0.001

**Figure 6.**
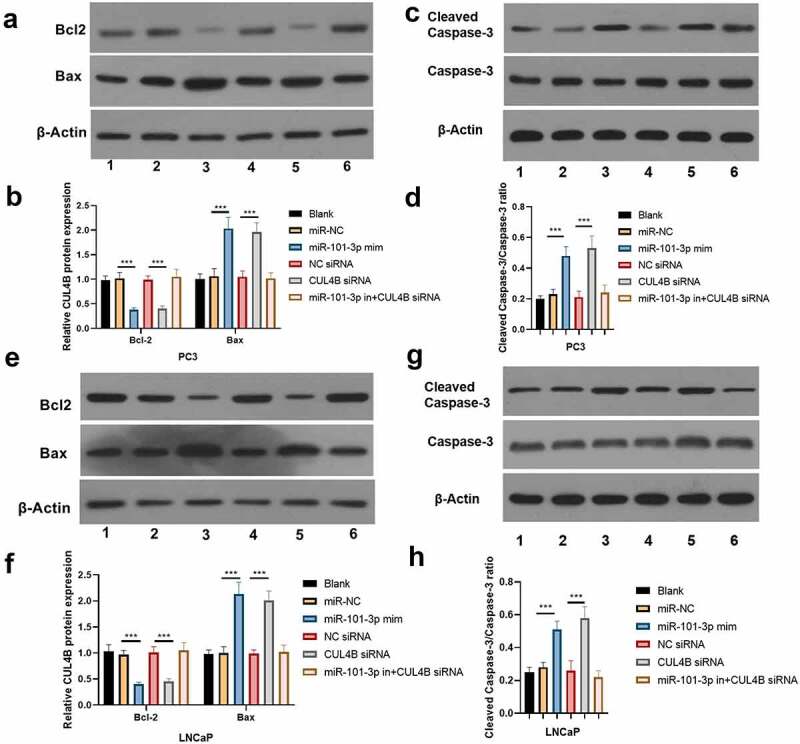
Western blotting approaches were adopted to detect proteins related to apoptosis (cleaved Caspase-3/Caspase-3, Bcl-2 and Bax) expression in LNCaP cells and PC3 cells. Noting: 1. the blank groups, 2. the miR-negative control groups, 3. the miR-101-3p simulation groups, 4. the Negative Control SiRNA groups 5. the siRNA of CUL4B groups 6. siRNA of CUL4B plus the inhibitors of miR-101-3p groups ***P < 0.001

The flow cytometry suggested that LNCaP as well as PC3 cellular apoptosis rate were significantly higher in miR-101-3p mimic groups than that in miR-NC group. On the contrary, the apoptosis rate was lower in the inhibitor plus CUL4B siRNA groups and NC siRNA groups than that of siRNA group (*P* < 0.001, Figure 5). Western Blot was used to detect the protein levels of Bcl-2, Bax and Cleaved Caspase-3/Caspase-3 (Figure 6). The results of Western Blot demonstrated that miR-101-3p increased the Bax and Cleaved Caspase-3/Caspase-3 ratio, whereas Bcl-2 expression was inhibited. In addition, the similar results were found in siRNA of CUL4B groups (*P* < 0.001). Nonetheless, miR-101-3p inhibitor reversed changes mentioned above through siRNA of CUL4B.

### 3.6. MiR-101-3p inhibited PCA cellular invasion as well as migration through targeting CUL4B

**Figure 7.**
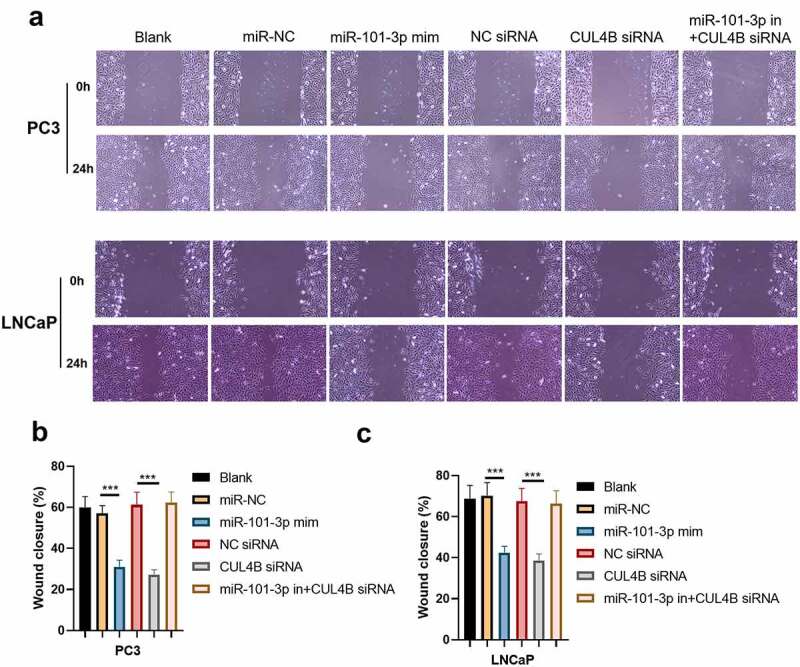
miR-101-3p inhibited Pca cellular migration through aiming for CUL4B. (a–c) The wound-healing experiments observed cellular migration in different groups. The closure rates (%) in scratch areas from different groups of LNCaP and PC3 cells were compared. ***P < 0.001

**Figure 8.**
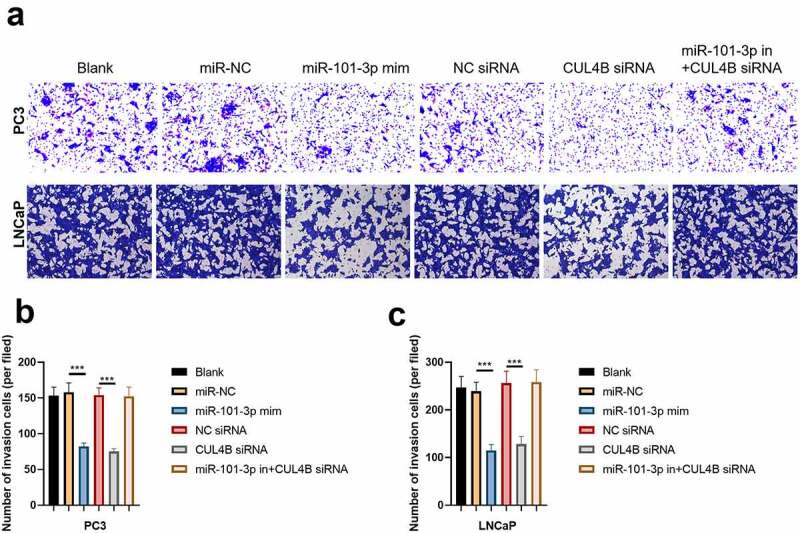
miR-101-3p suppressed Pca cellular invasion through aiming for CUL4B. (a–c) Transwell invasion experiments were used to observe invasion ability in LNCaP cells and PC3 cells. ***P < 0.001

We conducted scratch-healing experiments (Figure 7) and transwell invasion experiments (Figure 8) on LNCaP cells and PC3 cells to detect cellular migration and invasion abilities in vitro. The over-expression and decrease in CUL4B lessened cellular migration and invasion abilities. Closure percentage in scratch areas and cellular invasion numbers declined (*P* < 0.001). However, no evident differences existed in siRNA negative Control groups, miR-NC group, siRNA of CUL4B and miR-101-3p inhibitor groups in terms of cellular invasion and migration abilities (*P* > 0.05). There was no statistical difference among miRNAs of NC groups, miR-NC groups and siRNA of CUL4B plus miR-101-3p inhibitors groups in terms of cellular invasion and migration abilities (*P* > 0.05).

### 3.7. PI3K/AKT/mTOR pathway in PCA cells was halted through miR-101-3p targeting CUL4B

**Figure 9.**
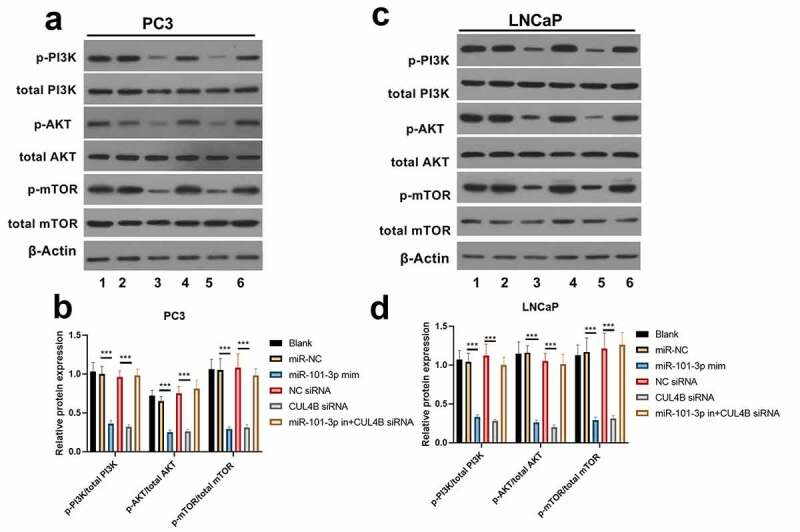
Western blotting methods were used to detect protein expression related to PI3K/AKT/mTOR signal in LNCaP as well as PC3 cells from different groups. Noting: 1. the blank groups, 2. the miR-negative control groups, 3. the miR-101-3p simulation groups, 4. the negative control SiRNA groups 5. the siRNA of CUL4B groups 6. siRNA of CUL4B plus the inhibitors of miR-101-3p groups ***P < 0.001

As shown in Figure 9, miR-101-3p mimic simulation as well as siRNA of CUL4B repressed PI3K/AKT/mTOR pathway in PC3 as well as LNCaP cells and they decreased p-AKT/total AKT, p-mTOR/total mTOR and p-PI3K/total PI3K expression (*P* < 0.001). Nonetheless, miR-101-3p inhibitor reversed the effects of siRNA of CUL4B.

### 3.8. Inhibition of CUL4B further enhanced the inhibitory effect of up-regulated miR-101-3p on PCA progression

**Figure 10.**
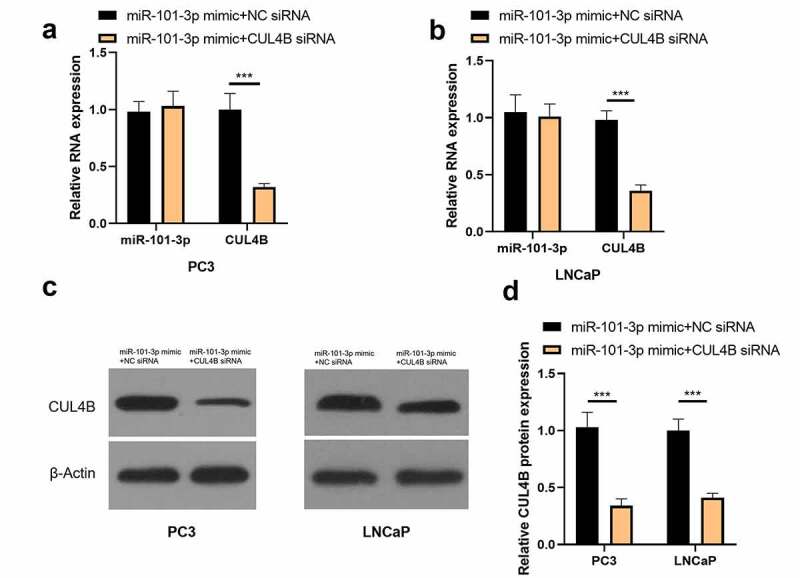
Verification of transfection efficiency of miR-101-3p and CUL4B. (a–b) qPCR to detect miR-101-3p and CUL4B expressions in PCA cells. (c–d) Western Blot to detect miR-101-3p and CUL4B expressions in PCA cells. ***P < 0.001

**Figure 11.**
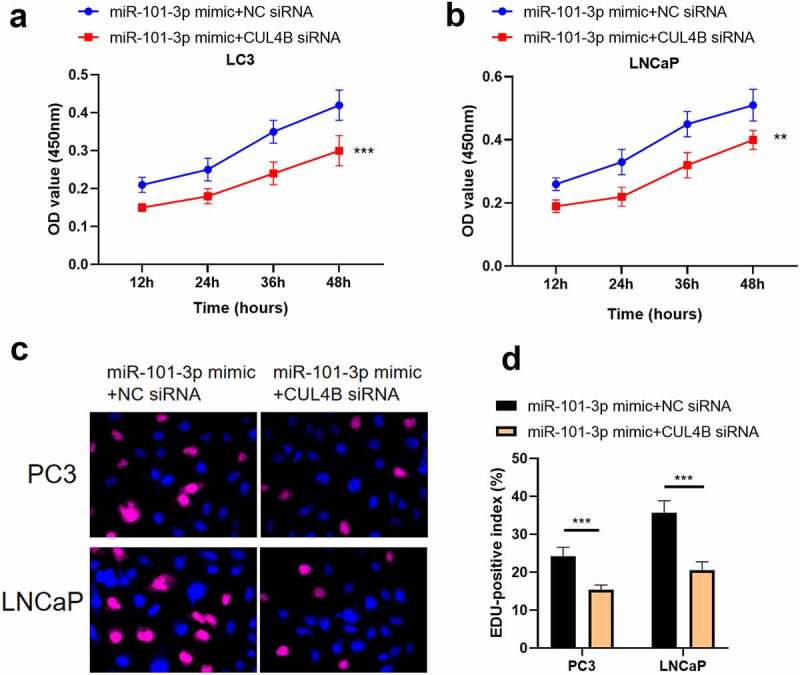
Inhibition of CUL4B further enhances the inhibitory effect of up-regulated miR-101-3p on PCA cell proliferation. (a–b) MTT approaches were used to detect LNCaP and PC3 cellular proliferation from different groups. C The inverted fluorescence microscope observed growth situation of LNCaP and PC3 cells. Cellular nuclei were dyed through DAPI staining (blue). Red dye meant that cells were proliferating. (d) PC3 cells were compared with LNCaP cells in terms of EDU positive index. The positive number in terms of EDU was shown in the form of the percentage number, which was a positive number/total cellular number in details. ***P < 0.001

**Figure 12.**
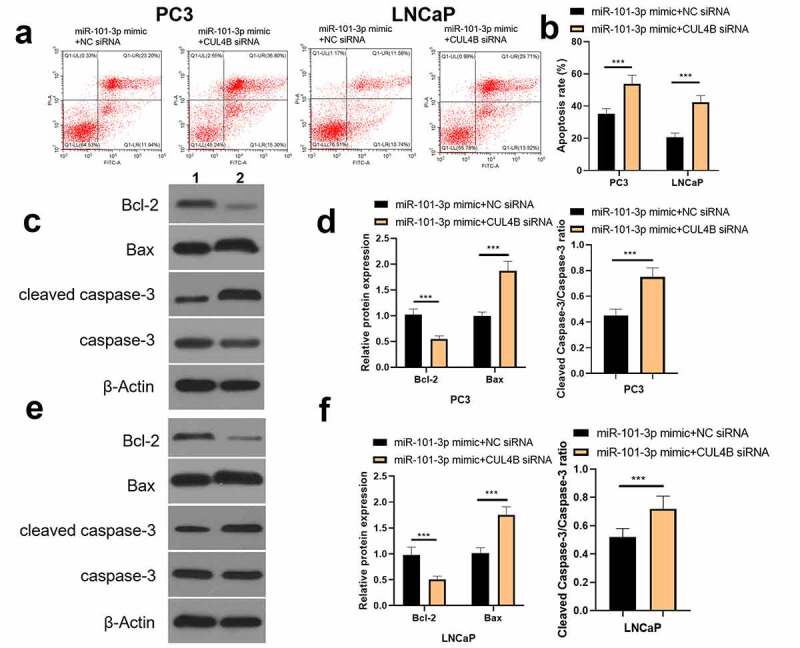
Inhibition of CUL4B further enhances the inhibitory effect of up-regulated miR-101-3p on PCA cell apoptosis. (a–b) We employed Annexin V-FITC/PI dye to display cellular apoptosis situation in LNCaP and PC3 cells. (c–f) Western blotting approaches were adopted to detect proteins related to apoptosis(Cleaved Caspase-3/Caspase-3, Bcl-2 and Bax) expression in LNCaP cells and PC3 cells.Noting:1. the miR-101-3p mimic+NC siRNA groups, 2. the miR-101-3p mimic+CUL4B siRNA groups,***P < 0.001

**Figure 13.**
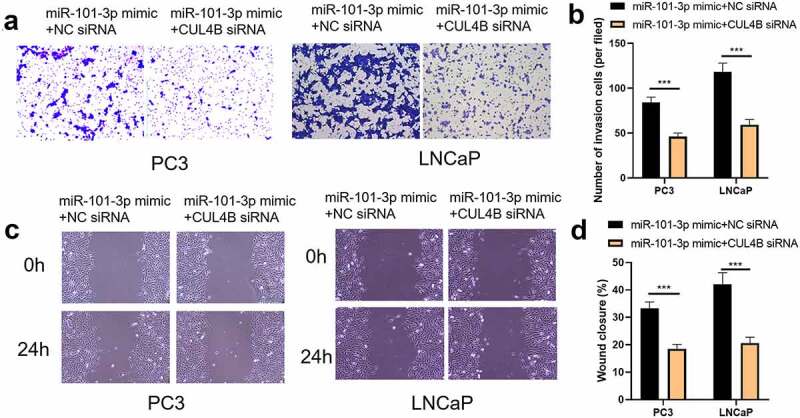
Inhibition of CUL4B further enhances the inhibitory effect of up-regulated miR-101-3p on PCA cell migration and invasion. (a–b) The wound-healing experiments observed cellular migration in different groups. The closure rates (%) in scratch areas from different groups of LNCaP and PC3 cells were compared. (c–d) Transwell invasion experiments were used to observe invasion ability in LNCaP cells and PC3 cells. ***P < 0.001

**Figure 14.**
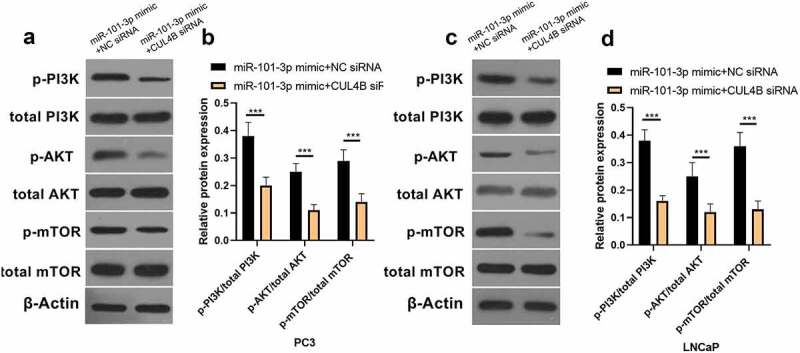
Western blotting methods were used to detect protein expression related to PI3K/AKT/mTOR signal in LNCaP as well as PC3 cells from different groups. ***P < 0.001

In order to further explore the axial relationship between miR-101-3p and CUL4B, we further knocked down CUL4B in cells transfected with miR-101-3p mimic. And qPCR and Western Blot were employed to detect that mRNA and protein expression of CUL4B were significantly decreased (Figure 10, P < 0.001). Next, through CCK8 and EDU experiments, it was found that compared with the miR-101-3p mimic+NC siRNA group, the proliferation ability of the miR-101-3p mimic+CUL4B siRNA group was significantly reduced (Figure 11, P < 0.001). Flow cytometry and Western Blot found that inhibition of CUL4B further enhanced the inhibitory effect of up-regulated miR-101-3p’s on PCA apoptosis (Figure 12, P < 0.001). In Figure 13, it was found that the migration and invasion ability of miR-101-3p mimic+CUL4B siRNA group was further inhibited. Finally, CUL4B siRNA also further inhibited the PI3K/AKT/mTOR pathway in PC3 and LNCaP cells (Figure 14, P < 0.001).

## 4. Discussion

As the second most common reasons relating to death from cancers in males, PCA is still regarded as the most common malignant cancers bothering males even though its incidence and mortality continuously decline [20]. People should try hard to deepen their understanding about PCA. Many researchers paid the emphasis on miRNAs in modifying mechanism of malignant cancers pathogenesis especially including miR-26a and miR-128 during PCA [21,22]. In this study, our findings offer the convincing evidence that implied miR-101-3p might be an anti-tumor factor for PCA. PCA cellular invasion, migration and proliferation could be halted through miR-101-3p inhibiting PI3K/AKT/mTOR signaling pathway by aiming for CUL4B.

We chose human PCA cellular system (LNCaP and PC3) to conduct the experiments in vitro. The results displayed that PCA cellular invasion, migration and proliferation could be prominently ceased by siRNA of CUL4B. It could promote its apoptosis, which was in great agreement with the paper that reached the same conclusion. Besides, it was found that CUL4B could mediate the PI3K/AKT/mTOR pathway. So it could exert some effects in terms of PCA. For instance, ZEB1-AS1 mediated the PI3K/AKT/mTOR pathway through miR-342-3p/CUL4B axis. ZEB1-AS1 modified PCA progression through miR-342-3p/CUL4B axis playing one role in PI3K serious pathway, which offered possible tactics for PCA therapy [23]. In this paper, all the expressions of p-mTOR, p-AKT as well as p-PI3K declined after CUL4B inhibition, indicating this pathway was suppressed. Some evidence said that CUL4B up-regulation in tumor cells might be regulated through miRNAs. miRNA-708 might halt osteogenic sarcoma progression through aiming for CUL4B [24]. Our research found that miR-101-3p expressed less in cancer tissues compared with that from other adjacent tissues, implying that for PCA tissues, little miR-101-3p expressed among PCA tissues than that from normal peripheral tissues. What attracted us was that the mutation of guanine to cytosine in 3ʹ-UTR from CUL4B damaged combination with miR-101-3p, causing over-expression of CUL4B. Those data displayed that there were some possibilities of miR-101-3p playing a role in translation modification in terms of CUL4B. Besides, we forecast that the combination site exists in 3ʹ-UTR of CUL4B via online website and dual-luciferase reporter experiments affirmed our assumption.

EDU and MTT staining results showed that the simulation had noticeable inhibition effects on human PCA cellular proliferation, which was similar to the outcomes from Doctor Zhang and other people. These outcomes suggested that inhibition effects from the simulation existed in human PCA cellular proliferation [25]. The downregulation of miR-101-3p might induce metastasis. We identified that it induced PCA cellular invasion as well as migration. The potential explanation was that miR-101-3p could halt angiogenesis in tumor cells while angiogenesis could not only provide the nutrition as well as excretion, but also transport tumor cells to the hosts, inducing their growth as well as metastasis. Besides, we found that miR-101-3p could promote LNCaP as well as pc3 cellular apoptosis. There are two crucial modification genes we have already known during the process of cellular apoptosis regulation-gene bax and gene bcl-2 [26]. The family bcl-2 can regulate cyto-c releasing to medicate caspase activation through cyto-c [27]. caspase3 is the most vital apoptosis executive proteins [28]. This study improved that apoptosis protein bax and cracking caspase-3 expression could be up-regulated through the help of miR-101-3p, which was consistent with results from Yong Lin and other researchers. And miR-101-3p could halt the PI3K/AKT/mTOR pathway in LNCaP and PC3 cells. The outcomes mentioned above suggested that miR-101-3p exerted the anti-cancer role in PCA through aiming for CUL4B.

## 5. Conclusion

In conclusion, our clinical outcomes displayed that PCA patients possessed less miR-101-3p and more CUL4B. The experiment in vitro indicated that PCA cellular migrating, invasion and proliferating were inhibited through miR-101-3p inducing PCA cellular apoptosis via aiming for CUL4B, which might be related with PI3K/AKT/mTOR suppression, providing a new perspective for the pathogenesis of PCA, and at the same time a promising target for the clinical treatment of PCA.

## Research highlights

1. miR-101-3p expression is low in prostate cancer, while CUL4B expression is high;
2. Up-regulated miR-101-3p of inhibited CUL4B suppressed prostate cancer cells.
3. miR-101-3p targets CUL4B to inhibit its expression;
4. This work provides reference for mechanism of treating prostate cancer.

## References

[1] Bray F, Ferlay J, Soerjomataram I, et al. Global cancer statistics 2018: GLOBOCAN estimates of incidence and mortality worldwide for 36 cancers in 185 countries. CA Cancer J Clin. 2018;68:394. 30207593 10.3322/caac.21492

[2] Patel SA, Vanharanta S. Epigenetic determinants of metastasis. Mol Oncol. 2017;11:79. 27756687 10.1016/j.molonc.2016.09.008 PMC5423227

[3] Gasnier A, Parvizi N. Updates on the diagnosis and treatment of prostate cancer. Br J Radiol. 2017;90:20170180. 28555500 10.1259/bjr.20170180 PMC5594995

[4] Barry MJ, Simmons LH. Prevention of prostate cancer morbidity and mortality: primary prevention and early detection. Med Clin North Am. 2017;101:787. 28577627 10.1016/j.mcna.2017.03.009

[5] Dall’Era MA, Cooperberg MR, Chan JM. Active surveillance for early-stage prostate cancer: review of the current literature. Cancer. 2008;112:1650. 18306379 10.1002/cncr.23373

[6] Pishgar F, Ebrahimi H, Moghaddam SS, et al. Global, regional and national burden of prostate cancer, 1990 to 2015: results from the global burden of disease study 2015. J Urol. 2018;199:1224. 29129779 10.1016/j.juro.2017.10.044

[7] Huang L, Chaoquan H, Cao H, et al. MicroRNA-29c increases the chemosensitivity of pancreatic cancer cells by inhibiting USP22 mediated autophagy. Cell Physiol Biochem. 2018;47:747–758. 29807360 10.1159/000490027

[8] Takayama K-I, Misawa A, Inoue S. Significance of microRNAs in androgen signaling and prostate cancer progression. Cancers (Basel). 2017;9(8):102. 10.3390/cancers9080102 PMC5575605 28783103

[9] Jin Q, He W, Chen L, et al. MicroRNA-101-3p inhibits proliferation in retinoblastoma cells by targeting EZH2 and HDAC9. Exp Ther Med. 2018 Sep;16(3):1663–1670. 30186385 10.3892/etm.2018.6405 PMC6122260

[10] Li B, Xie D, Zhang H. MicroRNA-101-3p advances cisplatin sensitivity in bladder urothelial carcinoma through targeted silencing EZH2. J Cancer. 2019;10:12. 10.7150/jca.33117 PMC6584933 31258770

[11] Li L, Shao MY, Zou SC, et al. MiR-101-3p inhibits EMT to attenuate proliferation and metastasis in glioblastoma by targeting TRIM44. J Neurooncol. 2019;141(1). DOI:10.1007/s11060-018-2973-7 30539341

[12] Jackson S, Xiong Y. CRL4s: the CUL4-RING E3 ubiquitin ligases. Trends Biochem Sci. 2009;34:562. 19818632 10.1016/j.tibs.2009.07.002 PMC2783741

[13] Vulto-van Silfhout AT, Nakagawa T, Bahi-Buisson N. Variants in CUL4B are associated with cerebral malformations. Hum Mutat. 2015;36:106. 25385192 10.1002/humu.22718 PMC4608231

[14] Miao C, Chang J, Zhang G. CUL4B promotes the pathology of adjuvant-induced arthritis in rats through the canonical Wnt signaling. J Mol Med (Berl). 2018;96:495. 29626254 10.1007/s00109-018-1635-8

[15] Fang ZZ, Wang M, Yuan Y, et al. miR-381 and miR-489 suppress cell proliferation and invasion by targeting CUL4B via the Wnt/betacatenin pathway in gastric cancer. Int J Oncol. 2019;54:733. 30483755 10.3892/ijo.2018.4646

[16] Wang X, Chen Z. Knockdown of CUL4B suppresses the proliferation and invasion in non-small cell lung cancer cells. Oncol Res. 2016;24: 271. 27656838 10.3727/096504016X14666990347473 PMC7838745

[17] Qi MH, Cui J, Jiao Y, et al. CUL4B promotes prostate cancer progression by forming positive feedback loop with SOX4. Oncogenesis. 2019;8:23. 30872583 10.1038/s41389-019-0131-5 PMC6418142

[18] Chen Z, Shen B-L, Qing-Ge F, et al. CUL4B promotes proliferation and inhibits apoptosis of human osteosarcoma cells. Oncol Rep. 2014;32:2047. 25189186 10.3892/or.2014.3465

[19] Alzahrani AS. PI3K/Akt/mTOR inhibitors in cancer: at the bench and bedside. Semin Cancer Biol. 2019 Dec 59:125–132. 31323288 10.1016/j.semcancer.2019.07.009

[20] Abdelrahman AE, Arafa SA, Ahmed RA. Prognostic value of Twist-1, E-cadherin and EZH2 in prostate cancer: an immunohistochemical study. Turk Patoloji Dergisi, 2017;33(3) 10.5146/tjpath.2016.01392 28832071

[21] Jin M, Zhang T, Liu C, et al. miRNA-128 suppresses prostate cancer by inhibiting BMI-1 to inhibit tumor-initiating cells. Cancer Res. 2014;74:4183–4195. 24903149 10.1158/0008-5472.CAN-14-0404 PMC4174451

[22] Jin M, Zhang T, Liu C, et al. MiRNA 26a expression in a novel panel of African American prostate cancer cell lines. Ethn Dis. 2010;20:S1–96–100. PMC3118047 20521394

[23] Ma T, Chen H, Wang P, et al. Downregulation of lncRNA ZEB1-AS1 represses cell proliferation, migration, and invasion through mediating PI3K/AKT/mTOR signaling by miR-342-3p/CUL4B axis in prostate cancer. Cancer Biother Radiopharm. 2020. DOI:10.1089/cbr.2019.3123 32275162

[24] Chen G, Zhou H. MiRNA-708/CUL4B axis contributes into cell proliferation and apoptosis of osteosarcoma. Eur Rev Med Pharmacol Sci. 2018;22(17):5452–5459. 10.26355/eurrev_201809_15805 30229816

[25] Zhang S, Ding L, Gao F, et al. Long non-coding RNA DSCAM-AS1 upregulates USP47 expression through sponging miR-101-3p to accelerate osteosarcoma progression[J]. Biochemistry and Cell Biology. 2020;98. 10.1139/bcb-2020-0031 32379981

[26] Song W, Song C, Chen Y, et al. Polysaccharide induced apoptosis in H22 cells through G2/M arrest and BCL2/BAX caspase-activated Fas pathway. Cell Mol Biol (Noisy-le-Grand). 2015;61(7):88–95. 26612738

[27] Gao G, Dou QP. N-terminal cleavage of bax by calpain generates a potent proapoptotic 18-kDa fragment that promotes bcl-2-independent cytochrome C release and apoptotic cell death. J Cell Biochem. 2000;80(1):53–72. 11029754 10.1002/1097-4644(20010101)80:1<53::aid-jcb60>3.0.co;2-e

[28] Freire R, Di Fagagna FD, Wu L, et al. Cleavage of the Bloom’s syndrome gene product during apoptosis by caspase-3 results in an impaired interaction with topoisomerase IIIalpha. Nucleic Acids Res. 2001;29(15):3172–3180. 11470874 10.1093/nar/29.15.3172 PMC55826

